# Left atrial thrombus attached to the orifice of the left superior pulmonary vein: a case report

**DOI:** 10.1007/s12574-024-00644-0

**Published:** 2024-02-10

**Authors:** Kazuhiro Nomura, Yuna Shinohara, Yoshie Nakajima, Shiro Iwanaga, Takuya Maeda, Keiji Yamamoto

**Affiliations:** 1 Department of Laboratory Medicine, Saitama Medical University Hospital, 38 Morohongo, Moroyama-machi, Iruma-gun, Saitama, 350-0495 Japan; 2 Department of Cardiovascular Medicine, Saitama Medical University Hospital, 38 Morohongo, Moroyama-machi, Iruma-gun, Saitama, 350‑0495 Japan; 3 Department of Cardiology, Saitama Medical University International Medical Center, 1397-1 Yamane, Hidaka, Saitama 350-1298 Japan

## Case

**Fig. 1:**
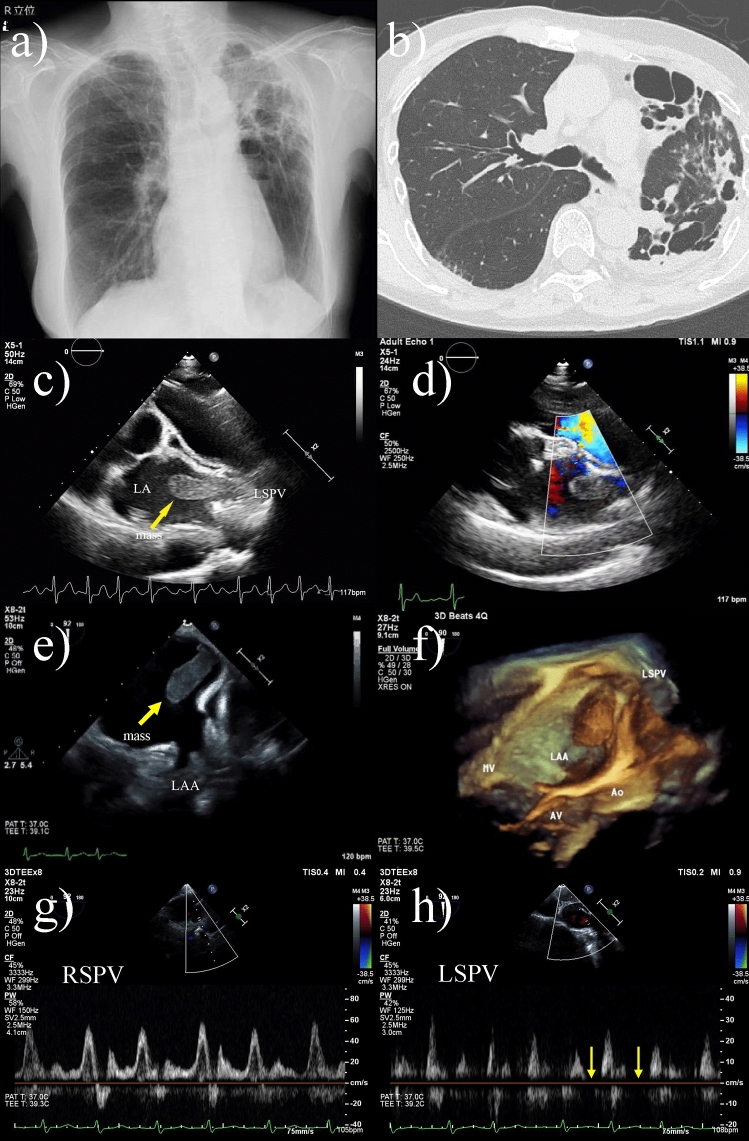
**a** Chest radiograph showed a cardiothoracic ratio of 49% and no cardiac enlargement, with abnormal shadows mainly in the left superior lung field and decreased air content in the left lung. **b** Chest computed tomography showed destruction of the structures of the left superior lobe of the lung. **c** Transthoracic echocardiography shows a mass protruding from the left superior pulmonary vein (LSPV) to the left atrium (LA) (arrows). The mass measures 32 × 12 mm and has a well-defined margin and an isoechoic and heterogeneous inside structure. **d** Color Doppler imaging confirms that there is no blood flow signal inside the mass. **e** Transesophageal echocardiogram (TEE) shows a mass (29 × 11 mm) attached to the LA wall at the LSPV orifice (arrows). **f** 3D-TEE showed that the thrombus adhered to a site distinctly different from the left atrial appendage, and was observed to be club-shaped from the LSPV to the LA. **g**, **h** Normal pulmonary vein blood flow is continuous, but the LSPV blood flow in this case is intermittent, with periods of cessation of blood flow (arrows). *MV* mitral valve, *AV* aortic valve, *Ao* aorta, *RSPV* right superior pulmonary vein

An 82-year-old woman presented with exertional dyspnea that developed following a persistent low-grade fever of 37.5 °C for 10 days. She had been diagnosed with *Mycobacterium avium* pulmonary infection 24 years earlier and had intermittently received the standard antibiotics combination therapy. On admission, blood test results showed mildly elevated tumor markers with CEA and CA 125 of 5.2 ng/mL (ref: 0–5.0 ng/mL) and 39 U/mL, (ref: 0–35.0 U/mL) respectively. and elevated d-dimer of 5.04 μg/ml. An electrocardiogram confirmed atrial fibrillation (AF) with an average heart rate of 121/min, which had not been observed during a routine examination 2 months earlier. Chest radiographs showed abnormal opacity and decreased air volume, mainly in the superior left lung field, where computed tomography also showed destruction of the structures of the left superior lobe of the lung (Fig. 1a, b).

Transthoracic echocardiography (TTE) showed a left ventricular ejection fraction of 60%, normal left ventricular wall motion, and no significant left heart valvular dysfunction. Additionally, TTE also showed dilation of the left atrium (LA; volume index, 35.5 mL/m^2^) and a mobile mass-like structure within the LA, whereas no obvious structures were found within the left atrial appendage (LAA). To observe the structure in more detail, we performed observation from a higher intercostal space. The structure appeared to be protruding from the left superior pulmonary vein (LSPV) into the LA (Fig. 1c, d, Movie 1). It was still difficult to identify the site of attachment, and thrombus, myxoma, and metastatic tumor were considered as differential diagnoses.

At this time, she was started on treatment with apixaban and bisoprolol for tachycardic AF (CHA_2_DS_2_-VASc score 2). On day 19, TEE showed that the mass attached to the LA wall near the LSPV orifice (Fig. 1e). On 3D-TEE, the structure was observed to be club-shaped from the LSPV to the LA (Fig. 1f, Movie 2, 3). The mass eventually shrank and disappeared with anticoagulation by day 99, and we finally diagnosed it as thrombus (Movie 4).

## Discussion

AF is the most common arrhythmia associated with stroke and embolism, and intra-atrial thrombi occurring in the LAA are the most frequently reported [1, 2]. Normal pulmonary venous blood flows continuously enter the LA, but in this case, in addition to AF tachycardia, tissue destruction in the left upper lobe reduced the return blood flow from the LSPV, causing a period of intermittent cessation of blood flow (Fig. 1g, h). As a result, the blood flow from the LSPV did not merge with the other three pulmonary venous blood flows in the LA and stagnated near the LA orifice of the LSPV, which may have led to thrombus formation outside the LAA. Pulmonary vein thrombosis is associated with early postoperative lobectomy, lung transplantation, and malignancy [3, 4], and thrombus formation at the LSPV stump after left upper lobectomy appears to have a similar pathogenesis [5].

## Conclusion

This report described a rare case in which a thrombus is attached to the orifice of the LSPV, suggesting the importance of recognizing intra-atrial thrombus formation outside the LAA on TTE and TEE.

## Supplementary Information

Below is the link to the electronic supplementary material.

Supplementary file1 Movie 1 Transthoracic echocardiography shows a mobile mass (32 × 12 mm) protruding from the left superior pulmonary vein to the left atrium (MP4 1628 KB)

Supplementary file2 Movie 2 3D-transesophageal echocardiogram showed that the thrombus adhered to a site distinctly different from the left atrial appendage, and was observed to be club-shaped from the left superior pulmonary vein to the left atrium. MV, mitral valve; AV, aortic valve; Ao, aorta (MP4 30502 KB)

Supplementary file3 Movie 3 3D-transesophageal echocardiogram showed that the thrombus adhered to a site distinctly different from the left atrial appendage, and was observed to be club-shaped from the left superior pulmonary vein to the left atrium. MV, mitral valve; AV, aortic valve; Ao, aorta (MP4 33130 KB)

Supplementary file4 Movie 4 Transthoracic echocardiography showed that the thrombus tended to regress with anticoagulation (MP4 101901 KB)

## Abbreviations

PT: Prothrombin time
AF: Atrial fibrillation
TTE: Transthoracic echocardiography
LA: Left atrium
LAA: Left atrial appendage
LSPV: Left superior pulmonary vein
TEE: Transesophageal echocardiogram
